# Restoring Gut-brain Function by Medicinal Herbs Offering Neuroprotection through Suppressing Inflammatory Pathways: A Systematic Review

**DOI:** 10.2174/011570159X353541250128101649

**Published:** 2025-06-02

**Authors:** Fatemeh Abbaszadeh, Sajad Fakhri, Behrang Shiri Varnamkhasti, Seyed Zachariah Moradi, Mohammad Hosein Olfati, Zanyar Moradi, Mohammad Reza Khirehgesh, Haroon Khan

**Affiliations:** 1 Neurobiology Research Center, Institute of Neuroscience and Cognition, Shahid Beheshti University of Medical Sciences, Tehran, Iran;; 2 Pharmaceutical Sciences Research Center, Health Institute, Kermanshah University of Medical Sciences, Kermanshah, 6734667149, Iran;; 3 Department of Pharmacy, Abdul Wali Khan University Mardan, Mardan, 23200, Pakistan;; 4 Department of Pharmacy, Korea University, Sejong, 20019, South Korea

**Keywords:** Medicinal herbs, neurodegenerative diseases, gut-brain axis, inflammatory pathway, neuroprotection, pharmacology

## Abstract

**Introduction:**

Neurodegenerative diseases (NDDs) refer to a progressive degeneration of the nervous system and are on the rise. Researchers are trying to reveal the crucial mechanisms behind NDDs to find novel therapeutic candidates with higher efficacy and lower side effects. Increasing evidence highlights the auspicious role of inflammatory mechanisms in the pathogenesis of NDDs.

**Methods:**

Based on the Preferred Reporting Items for Systematic Reviews and Meta-Analysis (PRISMA) guideline, a systematic and comprehensive review was done to evaluate the effect of medicinal herbs in restoring gut-brain function and anti-inflammatory mechanisms in combating neuroprotection. The electronic databases, including Scopus, PubMed, and ScienceDirect, were searched for the literature review. The manual search of reference lists and citations was also employed falling within the authors’ expertise.

**Results:**

As with other mechanisms, the bidirectional communication between the brain and gut, known as the gut-brain axis, has emerged as a potential target for therapeutic interventions. Since the gut-brain axis covers multiple mechanisms, especially inflammatory mechanisms in NDDs, it urges the need for finding novel multi-targeting agents. Medicinal herbs, with their rich repertoire of natural products, are multi-targeting candidates in combating several diseases. In this systematic and comprehensive review, we explore the potential of medicinal herbs in restoring gut-brain function and promoting neuroprotection by suppressing inflammatory pathways. Novel delivery systems and clinical applications of medicinal herbs are also highlighted to drawback the pharmacokinetic limitation in regulating the gut-brain axis-associated NDDs.

**Conclusion:**

Medicinal herbs provide neuroprotective responses through the modulation of gut-brain function and related inflammatory mediators.

## 1 INTRODUCTION

In recent decades, neurodegenerative diseases (NDDs) have been emerging concerns worldwide. Several pathophysiological mechanisms are behind neurodegeneration, including those of apoptosis, oxidative stress, and inflammation [1]. Neuroinflammation, characterized by chronic inflammation in the nervous system, is a common pathological feature observed in various NDDs. This inflammatory response is driven by activated immune cells and results in the release of pro-inflammatory molecules, leading to neuronal damage and cognitive impairment [2]. Additionally, there has been a growing understanding of the bidirectional communication between the gut and the brain, known as the gut-brain axis. This intricate axis network involves the exchange of signals and molecules between the gut microbiota, the collection of microorganisms residing in the gastrointestinal tract, and the central nervous system (CNS). The gut microbiota has been found to play a crucial role in regulating immune responses and modulating inflammation [3]. Studies have shown that alterations in the composition and diversity of the gut microbiota can impact neuroinflammatory processes [4, 5]. Dysbiosis, an imbalance in the gut microbial community, has been associated with increased neuroinflammation and the progression of NDDs [3, 6]. Since multiple mechanisms are behind the gut-brain axis-related inflammation in NDDs, it urges the need for finding novel multi-targeting agents. Medicinal herbs, with rich historical roots and extensive knowledge of natural products, provide a vast array of potential therapeutic options. Traditional medicinal practices from different cultures have identified numerous plants, herbs, and natural compounds known for their potent anti-inflammatory properties. These medicinal herbs have been utilized for centuries to modulate immune responses and alleviate inflammation in various disease conditions [7, 8].

This is the first systematic and comprehensive review aimed at exploring the potential of medicinal herbs in restoring gut-brain function and promoting neuroprotection by suppressing inflammatory pathways. Clinical trials of medicinal herbs are also developed regarding the regulation of the gut-brain axis and associated inflammatory pathways.

## 2 METHODOLOGY

### 2.1 Gut-brain Axis

**Fig. (1):**
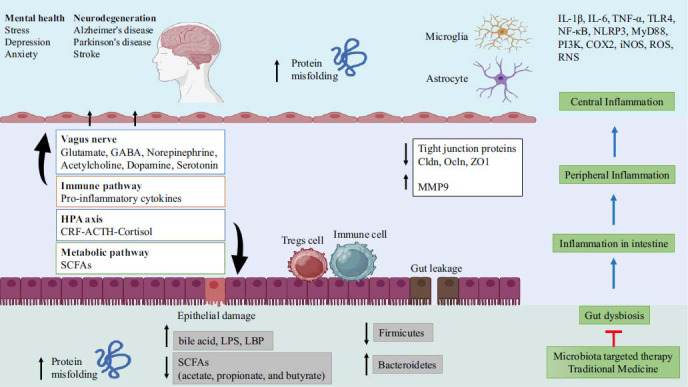
Proposed mechanisms of action of traditional medicine in the treatment of neurodegenerative diseases. Created with www.biorender.com.

The gut-brain axis is a complex and bidirectional communication system involving neural, endocrine, immune, and metabolic pathways. This connection allows the gut to influence cognition and mental health [9, 10]. As the largest crucial sensory-motor nerve, the vagus plays a crucial role in the gut-brain axis by facilitating bidirectional communication between the brain and the enteric nervous system (ENS) [11]. The vagus nerve carries information from the gut to the brain *via* afferent fibers. In turn, the brain sends signals back to the gut *via* efferent fibers, regulating various gut functions such as digestion, absorption, and gut motility. This bidirectional communication allows the brain to maintain homeostasis in the gut. In sum, the gut-brain axis consists of four major pathways that possess critical roles in NDDs [12]. These major factors are the neurologic pathway, hypothalamic-pituitary-adrenal (HPA) axis, immune pathway, and metabolic pathway (Fig. **1**).

### 2.2 Neurologic Pathway

The vagus nerve and the ENS work together to maintain homeostasis in the gut and regulate various physiological processes. The vagus nerve sends signals to the ENS, which then responds by regulating gut functions such as peristalsis and enzyme secretion. This communication helps to coordinate gut functions with higher brain functions, such as regulating appetite and satiety [13]. The vagus nerve is also involved in the production and release of neurotransmitters in the gut, which play important roles in regulating gut functions and influencing brain function. Accordingly, serotonin (5-HT), norepinephrine, dopamine, and gamma-aminobutyric acid (GABA) are neurotransmitters that are produced in the gut and influence gut motility and pain perception. Dysfunction in the gut-brain-related neurologic pathway has been linked to various NDDs [13, 14].

### 2.3 Hypothalamic-pituitary-adrenal (HPA) Axis

The HPA axis is a complex network involving the hypothalamus, pituitary gland, and adrenal glands, which play a key role in regulating the body’s stress response. Certain bacteria in the gut can produce neurotransmitters and neuropeptides that can directly impact the function of the HPA axis. Additionally, the gut microbiota can modulate the production of stress hormones (*e.g*., cortisol) by affecting the activity of involved enzymes [15]. Also, the gut microbiota can influence the permeability of the intestinal barrier, also known as “leaky gut”, which leads to the release of pro-inflammatory mediators. These molecules can then activate the immune system and trigger inflammatory responses, which can ultimately impact the function of the HPA axis [16].

### 2.4 Immune Pathway

The gut has its immune system, referred to as the gut-associated lymphoid tissue (GALT). The GALT contains immune cells that release cytokines and chemokines in response to the presence of pathogens or other stimuli. Such inflammatory mediators influence the brain by either directly or indirectly affecting neural activity [17]. Cytokines and chemokines can interact with various receptors present in neurons and glial cells, thereby modulating neuronal activity, synaptic plasticity, and neurotransmitter release, ultimately affecting various brain functions, including cognition and behavior. Also, they can influence the functional characteristics of astrocytes, microglia, and lymphocytes within the CNS, determining the fate of neurological disorders driven by neuroinflammation [18, 19].

### 2.5 Metabolic Pathway

The metabolic activities of gut microbiota have a significant impact on host metabolism, affecting energy balance and lipid metabolism. The production of short-chain fatty acids (SCFAs) through the fermentation of dietary fiber is a major metabolic pathway influenced by gut microbiota. SCFAs are absorbed by the host and serve as an energy source for intestinal cells [20]. Additionally, SCFAs have anti-inflammatory properties, regulate appetite, and influence glucose and lipid metabolism. Gut microbiota also plays a role in the metabolism of bile acids, which are essential for the digestion and absorption of dietary fats in preventing metabolic syndrome [21]. In addition, gut microbiota can impact the metabolism of cholesterol by converting cholesterol into different metabolites, such as SCFAs or coprostanol. The balance between cholesterol absorption and elimination is influenced by gut microbiota and impacts cholesterol levels in the body [22]. In addition to the regulatory role of gut microbiota on carbohydrates, bile acids, and cholesterol, gut microbiota can also influence the metabolism of various other metabolites, including amino acids, vitamins, and neurotransmitters [23]. Gut bacteria can produce certain vitamins, such as vitamin K and B vitamins, that are essential for human health. They can also metabolize dietary polyphenols, producing metabolites with potential health benefits. Finally, certain bacteria possess enzymes that can metabolize and modify xenobiotics, including drugs, environmental toxins, and dietary compounds. This can impact the bioavailability and toxicity of these substances [24, 25].

Altogether, regulating four major gut-brain pathways could play major roles in combating NDDs.

## 3 GUT-BRAIN AXIS AND ASSOCIATED NEUROINFLAMMATION IN NEURODEGENERATIVE DISEASES

Several key mechanisms have been identified that highlight the role of inflammation in NDDs *via* the gut-brain axis. Of major mechanisms, immune dysregulation, blood-brain barrier (BBB) permeability, microglia activation, and protein aggregation play critical roles.

### 3.1 Immune Dysregulation

The gut microbiota plays a crucial role in the development and maturation of the immune system in infants. Exposure to various microbes helps train the immune system to distinguish between harmful pathogens and harmless antigens, which is essential for the natural maturation of the intestine and immune system in infants [26]. The gut microbiota undergoes significant transformations during the first 1000 days after birth, driven by changes in diet and environmental factors. Disruptions to the microbiota during this window can have long-term effects on the infant's health, including the development of inflammatory and autoimmune disorders, neurological disorders, and obesity [27]. Two key pattern recognition receptor (PRR) systems, toll-like receptors (TLRs) and nucleotide-binding oligomerization domain molecules (NODs), are crucial in recognizing gut microbiota that is present in intestinal epithelial cells (IECs), macrophages, and dendritic cells (DCs). When microbes are detected, these receptors initiate intracellular signaling pathways, leading to the expression of various molecules like cytokines and chemokines, triggering pro-inflammatory and antimicrobial responses [17].

Certain types of bacteria found in the gut, specifically those belonging to the Firmicutes and Bacteroidetes phyla, can stimulate regulatory T cells (Tregs) through specific signaling pathways involving PRRs and their ligands, referred to as pathogen-associated molecular patterns (PAMPs) [28]. Additionally, these gut bacteria can impact Tregs by providing antigens that are processed and presented to Tregs by antigen-presenting cells (APCs). On the other hand, *Faecalibacterium prausnitzii* is a butyrate-producing bacterium in the gut microbiome [29]. Butyrate serves as an energy source for the cells lining the colon, helps maintain the integrity of the gut barrier and balance of gut bacteria, and enhances the function of Tregs [30]. This interaction results in the production of anti-inflammatory cytokines such as interleukin (IL)-10 and transforming growth factor-β (TGF-β) [31, 32], which play a crucial role in maintaining a balanced immune system by suppressing the activation and growth of pro-inflammatory T cells. This procedure could also inhibit the production of inflammatory molecules like tumor necrosis factor-alpha (TNF-α) and interferon-gamma (IFN-γ) and facilitate the transformation of naïve T cells into Tregs [29]. Peroxisome proliferator-activated receptor gamma (PPAR-γ) activation by butyrate supports a hypoxic environment favoring SCFA-producing bacteria while restraining pathogens. SCFAs mediate pathogen control through acidification, and limiting their growth advantages [17, 33].

In contrast, gut dysbiosis can increase the abundance of potentially pathogenic gram-negative bacteria such as Enterobacteriaceae. These bacteria can disrupt the epithelial barrier, increasing intestinal permeability and allowing the translocation of bacteria and their metabolic products across the mucosal barrier [34]. This disruption also increased susceptibility to infections by producing pro-inflammatory cytokines and altering B- and T-cell populations, further contributing to the development of inflammation. Also, they can activate immune cells, such as DCs and macrophages, then stimulate pro-inflammatory immune responses [17].

### 3.2 Blood-brain Barrier (BBB) Permeability

The gut microbiota influences BBB development and function. Studies have shown that the lack of normal gut flora in adult germ-free (GF) mice is associated with increased BBB permeability, both in the adult animal and in the embryos of GF mice [35].

SCFAs, such as acetate, butyrate, and propionate, produced by the gut microbiota through the fermentation of polysaccharides, have been found to affect BBB integrity. The binding of SCFAs to specific G-protein-coupled receptors (GPRs) called GPR41 and GPR43 on brain endothelial cells or their entry *via* transporters (MTC1, Fatty acid translocase/cluster of differentiation 36 (FAT/CD36)) triggers signaling cascades that regulate tight junction proteins [36]. This leads to a reduction in paracellular permeability and an enhancement of the integrity of the BBB. Butyrate inhibits histone deacetylase (HDAC), which results in the deacetylation of histones and non-histone proteins. This modulation plays a role in the hyperacetylation of PPARγ and its translocation into the nucleus, potentiating transcription of tight junction proteins and the maintenance of BBB integrity. SCFAs also activate the canonical Wnt/β-catenin pathway, which is important for the regulation of tight junction protein expression (Zonula occludens-1 (ZO-1), occludin, and claudin), to maintain BBB integrity. Moreover, SCFAs can modulate the levels of nuclear factor kappa B (NF-κB), nuclear factor erythroid 2-related factor 2 (Nrf2), and heme oxygenase (HO), which are involved in antioxidant defenses, mitochondrial function, and the regulation of redox homeostasis. This pathway helps protect brain endothelial cells against oxidative stress and inflammation [37].

During systemic inflammation and infection, the BBB becomes more permeable to solutes. This increased permeability allows toxins and immune cells to enter the brain, which can disrupt the normal functioning of the BBB [38]. Consequently, immune cells are activated, and various molecules such as cytokines, chemokines, adhesion molecules, cyclooxygenase-2 (COX-2) and inducible nitric oxide synthase (iNOS) enzymes [39, 40], nucleotide-binding and oligomerization domain (NOD)-like receptor protein 3 (NLRP3) inflammasome [41], and matrix metalloproteinase-9 [42] are released. These elements impact all components of the neurovascular unit, leading to further disruptions in BBB integrity and contributing to neuroinflammation [37].

### 3.3 Activation of Microglia

Microglia are indeed the primary immune cells in the CNS, responsible for surveillance, maintenance, and immune defense in the brain. They constantly monitor their environment for potential threats, such as pathogens or damaged cells, and play a crucial role in maintaining brain homeostasis [43].

In cases of dysbiosis-induced inflammation, inflammatory signals can be transmitted to the brain through various pathways, including the vagus nerve and circulating immune cells [44]. These signals can activate microglia and trigger an immune response in the brain. Activated microglia undergo morphological changes and release inflammatory mediators, including cytokines such as TNF-α, IL-1β, and IL-6. These cytokines play a significant role in initiating and propagating the inflammatory response. Furthermore, activated microglia also produce ROS and reactive nitrogen species (RNS), collectively known as free radicals. These molecules have the potential to cause oxidative stress and damage to surrounding neurons and other cells in the brain [45, 46]. Prolonged activation of microglia can lead to a chronic neuroinflammatory state, which is associated with various NDDs, including Alzheimer’s disease (AD), Parkinson’s disease (PD), and multiple sclerosis (MS). The sustained release of inflammatory mediators and free radicals can disrupt normal neuronal function and contribute to neuronal damage and degeneration [46].

### 3.4 Protein Aggregation

Protein misfolding is a process where proteins do not fold into their proper three-dimensional structures. In the case of NDDs, such as AD and PD, specific proteins like amyloid-beta (Aβ) and alpha-synuclein are prone to misfolding. These misfolded proteins tend to aggregate, forming clumps or aggregates [47]. A significant part of amyloids is produced by bacteria in the human gut. These amyloid proteins help bacteria attach to cells and form biofilms, as well as resist physical and immune stimuli. Bacterial amyloids can act like prion proteins and lead another amyloidogenic protein to acquire a pathogenic β-sheet structure. Various organisms have been found in the human microbiome, such as *Klebsiella*, *Streptococcus*, *Salmonella*, *Staphylococcus, Mycobacteria, Bacillus*, and *Citrobacter* species that produce extracellular amyloids. These amyloids can activate the innate immune system and trigger an inflammatory response (NF-κB, TLRs, iNOS) [48]. Gut microbiota may also influence inflammatory responses to amyloid deposits in the brain, potentially impacting cerebral damage. Consequently, exposure to bacterial amyloid proteins in the gut may activate the immune system, leading to an increased immune response to the formation of amyloid in the brain [48].

On the other hand, the presence of microorganisms and microbiota in the environment can trigger the accumulation of α-synuclein in the extracellular matrix, setting off a series of pathological events that result in oxidative stress, inflammation of the mucosal lining, and ultimately the formation of alpha-synuclein aggregates [49]. Notably, the abundance of butyrate-producing bacteria such as *Blautia*, *Coprococcus*, and *Roseburia*, as well as Prevotellaceae, a family of bacteria involved in the production of intestinal mucin and SCFAs from fiber fermentation in the colon, is diminished in individuals with PD [50, 51]. This reduction may lead to a decrease in the intestinal mucosa and an increase in intestinal permeability, making it easier for α-synuclein to cross the intestinal barrier and enter the ENS. This process could contribute to a sustained overproduction of α-synuclein or its misfolding [50].

Inflammation can exacerbate protein misfolding and aggregation in several ways. Firstly, inflammatory molecules can directly interact with proteins, altering their folding pathways and stability. This can lead to the formation of misfolded proteins [52]. Additionally, inflammation can disrupt the cellular machinery responsible for protein quality control, such as the chaperone proteins and proteasome system. This disruption can impair the clearance of misfolded proteins, allowing them to accumulate [53]. Furthermore, inflammation can promote the production of ROS and RNS, which are highly reactive molecules. These ROS and RNS can directly damage proteins, leading to their misfolding and aggregation [54]. Inflammatory processes can also compromise the BBB, allowing inflammatory molecules and immune cells to enter the brain. Once inside the brain, these molecules and cells can directly contribute to the aggregation of misfolded proteins [55]. The accumulation of misfolded proteins, particularly amyloid-beta and alpha-synuclein, can induce neurotoxicity. These aggregates can disrupt cellular processes, impair synaptic function, and induce neuronal death. The exact mechanisms by which misfolded proteins exert their toxic effects are still under investigation, but it is thought that they can trigger oxidative stress and inflammation and impair various intracellular signaling pathways [56].

In summary, the gut microbiota plays a crucial role in modulating inflammation, which influences the pathogenesis of NDDs. Immune dysregulation and systemic inflammation, activation of microglia, protein aggregation, and influence on the BBB are key mechanisms linking the gut microbiota, inflammation, and neurodegeneration. Understanding and targeting this gut microbiota-mediated inflammation could provide potential therapeutic avenues for the prevention and treatment of NDDs. Strategies to modulate the gut microbiota, such as dietary interventions (*i.e*., medicinal herbs), may help reduce inflammation and improve mental health and NDDs.

## 4 THE DYNAMIC ECOSYSTEM OF GUT MICROBIOTA: FACTORS INFLUENCING THE MEDICINAL HERB PROPERTIES

The composition of gut microbiota is not a fixed feature; rather, it is a dynamic and intricate ecosystem that evolves throughout an individual’s life. This complexity is shaped by a multitude of factors, both intrinsic and extrinsic, that influence the microbial communities residing in the gastrointestinal tract [57]. The implications of these factors on health are profound. A balanced gut microbiome is vital for numerous physiological processes, including nutrient metabolism, immune function, and protection against pathogens. Conversely, dysbiosis has been linked to a range of health issues, including neurodegenerative disease, obesity, inflammatory bowel disease, and metabolic disorders [58].

One of the most significant periods influencing gut microbiota composition occurs during early life. The establishment of these microbial communities starts at birth, where factors such as gestational age, the mode of delivery—vaginal or cesarean—and infant feeding practices, whether breastfeeding or formula feeding, play critical roles. These initial experiences not only set the groundwork for an individual’s unique microbiota profile but also have long-lasting implications for health [59, 60].

In addition to diet, lifestyle factors such as physical activity, stress levels, and personal habits also play essential roles in shaping gut microbiota. Regular exercise has been associated with a more diverse and resilient microbiome, while high-stress levels can lead to dysbiosis, resulting in an imbalance of microbial populations [61]. Medications, particularly the use of antibiotics, can significantly alter the composition of gut microbiota. While antibiotics are effective in combating infections, they can also reduce microbial diversity, leading to potential long-term health consequences [62]. Besides, environmental factors further complicate this dynamic landscape. Elements such as geographic location, cultural practices, and exposure to varying environments can all influence the diversity and stability of gut microbiota. For instance, individuals residing in rural areas often exhibit a more diverse gut microbiome compared to their urban counterparts, a difference attributed to variations in diet, lifestyle, and exposure to nature [63].

Diet is another powerful modulator of gut microbiota composition. Research has demonstrated that the food we consume can lead to rapid and significant changes in microbial diversity and abundance. For example, diets high in fats may favor the proliferation of specific bacterial strains linked to inflammation, while those rich in plant-based foods tend to enhance microbial richness and diversity [64, 65].

The impact of herbal medicines on gut health underscores the intricate relationship between dietary choices and microbial dynamics, as these natural remedies often contain bioactive compounds that can further influence microbial interactions within the gut. The medicinal properties of herbal medicines are influenced by a myriad of factors that can significantly affect their efficacy and therapeutic potential. One of the primary determinants of herbal efficacy is the quality of the plant source. Different species or varieties of the same plant may possess varying concentrations of active compounds, leading to differences in therapeutic outcomes. Furthermore, the conditions under which a plant is grown—such as soil quality, climate, and cultivation practices—can impact the concentration of beneficial phytochemicals. The timing of harvest also plays a crucial role; certain compounds may peak at specific growth stages, necessitating careful attention to the optimal harvest time to ensure maximum potency [66, 67].

Also, the preparation and processing methods used for herbal medicines are critical in determining their therapeutic value and quality. The choice of solvent used for extraction can significantly impact the bioavailability of active compounds in the final product [68]. Some compounds may be more soluble in alcohol, while others may be better extracted with water. Improper extraction can lead to the loss of active compounds or the inclusion of unwanted substances [67, 69]. Moreover, the formulation of herbal remedies, particularly in polyherbal combinations, can lead to synergistic effects that enhance efficacy or, conversely, antagonistic interactions that may diminish therapeutic benefits [69]. Proper storage conditions must also be maintained, as exposure to light, humidity, and temperature changes can degrade active compounds over time, reducing their effectiveness [70, 71].

Genetic variability and individual patient factors significantly influence the efficacy of herbal medicine and can affect how herbal compounds are metabolized. Some patients may process these compounds more quickly or slowly, impacting the effectiveness of the treatment. This variability can result in significant differences in how individuals respond to the same herbal remedy, necessitating a more personalized approach to herbal medicine [72]. Additionally, the presence of comorbidities can influence not only the safety profile of herbal remedies but also their effectiveness, as underlying health conditions may alter the body’s response to the treatment [73]. Lifestyle factors, such as diet and exercise, also play a role; a healthy lifestyle may enhance the effectiveness of herbal medicines, while poor lifestyle choices could diminish their potential benefits [74].

Promoting gut microbiota health requires a multifaceted approach that emphasizes a diverse, nutrient-rich diet, regular physical activity, effective stress management, and mindful use of medications, particularly antibiotics. Furthermore, the effectiveness of herbal medicines in supporting gut health is influenced by factors such as plant quality, preparation methods, and individual patient variability. A comprehensive understanding of these dynamics is crucial for developing targeted strategies to enhance gut health and prevent related diseases.

## 5 SEARCH STRATEGY

According to the PRISMA guideline, a comprehensive and systematic review was done to evaluate the effect of medicinal herbs in restoring gut-brain function and anti-inflammatory responses towards neuroprotection. Scholarly electronic databases, including PubMed, Scopus, and ScienceDirect, were employed for the literature search. All English language articles are included through May 30, 2024. The following keywords were employed for the search: (neuro* OR multiple sclerosis OR amyotrophic lateral sclerosis OR Huntington* OR spinal cord injury OR Alzheimer* OR Parkinson* OR brain) [title/abstract] AND (plant OR herb OR natural product OR phytochemical* OR alkaloid* OR polyphenol* OR terpene* OR flavonoid* OR coumarin* OR glucosinolate*) [title/abstract] AND (gut-brain) [title/abstract]. Accordingly, the screening is done based on including herbal medicine. Two independent authors (S.F. and S.Z.M.) designed and applied the search strategy, which was finalized by the senior author (H.K.).

Of the whole found 898 studies, 414 duplicated results, and 253 review articles were excluded. In this line, 112 articles were excluded according to their title/abstract, 76 articles were excluded according to their full-text information, and 12 non-English articles were omitted. Finally, 31 articles were directly included in this systematic review. However, based on the author’s expertise and checking reference lists of included studies, additional related articles were added. Fig. (**2**) presents the PRISMA flowchart on the literature search strategy and includes relevant studies.

## 6 RESULTS AND DISCUSSION

### 6.1 Regulatory Role of Medicinal Herbs on Gut-brain Axis-related Inflammatory Mechanisms in Combating NDDs

Medicinal herbs have shown major potential in modulating the gut-brain axis and associated inflammatory signaling pathways against neurodegenerative disorders, including Alzheimer's disease (AD), cognitive decline, Parkinson's disease (PD), stroke, and other NDDs.

#### 6.1.1 Alzheimer's Disease (AD) and Cognitive Decline

AD is the most common cause of dementia, characterized by the accumulation of Aβ in the brain and by memory loss, cognitive decline, and behavioral changes that worsen over time. The bacteria in the gut microbiota can produce high levels of amyloids and lipopolysaccharides, which may impact signaling pathways and the release of inflammatory cytokines associated with the progression of AD [75]. Systemic inflammation from factors like aging, infections, and chronic diseases can also contribute to AD pathology by increasing pro-inflammatory signals in the brain [76].

There is currently no cure for AD, but some traditional medicines and their compounds (Table **1**) have shown potential in managing symptoms, reducing neuroinflammation, protecting against neurodegeneration, modulation the gut microbiota, regulating the production of microbial metabolites, and improving cognitive function [77]. An experimental study provided evidence that Korean Red Ginseng (*Panax ginseng* Meyer) could improve AD pathology in mice by modulating the gut microbiota, specifically by increasing the dominance of Lactobacillus. This effect may be associated with a reduction in Aβ accumulation, higher expression of the tight junction protein Claudin-5, and a decrease in microglial activation [78]. Targeting Aβ protein aggregation and gut microbiota is also followed by other researchers. Following oral administration of *Triphala*, a polyphenol-rich Ayurvedic herbal mixture, for 60 days to 5XFAD mice, a model of AD, there was an improvement in cognitive function, decreased serum Aβ levels, and reduced mRNA expression of amyloid precursor protein in the brain. Additionally, there was an increase in anti-inflammatory and antioxidant activity in the serum, higher levels of disease-modifying bacteria such as Bacteroidetes and Verrucomicrobiota, and an increase in butyrate levels [79, 80]. In Asian countries, *Forsythiae Fructus* and Cassiae Semen are commonly used as herbal remedies to reduce inflammation. Research suggested that Forsythiae Fructus may be more effective than Cassiae Semen at preventing memory impairments. It did this by inhibiting the accumulation of Aβ, modulating the hippocampal pAkt/pGSK-3β/pFoxO1 pathway, reducing tau protein, increasing BDNF, and promoting the growth of beneficial bacteria [81].

*Coptis chinensis* Franch (CCF) is an intricate herb with a wide range of pharmacological properties and potential therapeutic applications. In a study using mice, CCF significantly improved cognitive dysfunction. Also, CCF affected histidine and phenylalanine metabolic pathways and increased the levels of acetic acid and indole-3-lactic acid in SCFAs in the serum and feces of mice [82]. As another study targeted metabolic pathways and SCFA, aqueous extract from *Codium Fragile* (AECF) demonstrated significantly improved spatial learning and memory performance in mice exposed to particulate matter. This extract also mediated the composition of the intestinal microbiome, elevating the production of SCFAs (acetate and propionate) and enhancing the expression of tight junction proteins such as claudin-1 and occluding in the gut. Additionally, AECF improved antioxidant systems in the colon and brain by raising levels of superoxide dismutase (SOD) and glutathione (GSH) while decreasing malondialdehyde (MDA) content. Furthermore, it reduced protein expression levels of TLR-4, MyD88, p-JNK, p-NF-κB, p-IκB-α, COX-2, TNF-α, Caspase1, IL-1β, Bax/Bcl-2 and enhanced mitochondrial function in brain tissues by boosting mitochondrial membrane potential (MMP) and decreasing ROS. In line, it was associated with an increase in the abundance of beneficial bacteria while decreasing the abundance of harmful ones [83].

Danggui Shaoyao San had a significant positive impact on learning and spatial memory in rats with AD by modulating various physiological processes. The formulation contained an enriched probiotic called Ligilactobacillus, which played a role in regulating several metabolites, including Ophthalmic acid, Phosphocreatine, Azacridone A, NAD, and Inosine. Danggui Shaoyao San was able to regulate purine and nicotinate-nicotinamide metabolism, restore balance in *Candidatus Saccharibacteria*-Ophthalmic acid interaction, and maintain gut microbiome-metabolite homeostasis [84, 85]. Furthermore, it reduced the expression of pro-inflammatory cytokines such as IL-1β, IL-6, and TNF-α in the hippocampus, which helped alleviate the inflammatory state. It also increased the levels of antioxidative enzymes such as SOD, CAT, and GSH-PX while decreasing the levels of MDA [85].

The cognitive impairment of mice induced with D-galactose/aluminum chloride (D-gal/AlCl3) was improved by *Rhizoma gastrodiae* water extract (WERG). The extract decreased the expression level of p-Tau (phosphorylated Tau) at threonine 231, which is associated with the accumulation of abnormal Tau and the formation of tangles. Additionally, WERG-H treatment was found to have a positive association with the abundance of beneficial gut bacteria [86].

By improving the expression of tight junction proteins (MUC-2, ZO-1, occludin, and claudin-1), the leaves of *Eucommia ulmoides* showed positive effects on cognitive dysfunction in mice with Dextran Sulfate Sodium (DSS)-induced colitis. Besides, the leaves increased acetic acid and butyric acid levels, decreased mitochondrial ROS, and improved mitochondrial MMP and ATP production. Moreover, the leaves reduced the protein expression levels associated with inflammation, such as TLR4, MyD88, COX-2, NF-κB, TNF-α, IL-1β, iNOS, and caspase-1 [87]. In another study targeting tight junction proteins of the gut-brain axis, *Acanthopanax senticosus* extract improved learning and memory in mice exposed to radiation by increasing the expression of BDNF and levels of important neurotransmitters like 5-HT, ACH, and NE in the hippocampus while decreasing GABA and NF-κB. It may also reduce inflammatory markers like TNF-α, IL-6, and IL-1β in the colon and hippocampus. Additionally, it could increase the expression of tight junction proteins such as claudin, ZO-1, and occludin and highlight the presence of Allobaculum, Peptostreptococcaceae, Clostridiales_vadinBB60_group, Ruminococcaceae, Streptococcus, and Coriobacteriaceae_UCG_002 [88]. As another regulator of tight junction proteins, Jiao-Tai-Wan, a traditional Chinese herbal formula composed of *Rhizome coptidis* and *Cortex cinnamomi*, demonstrated notable benefits in improving cognitive impairment and reducing inflammation in rats experiencing chronic partial sleep deprivation. These impacts are achieved by effectively lowering the amounts of Aβ_42_, caspase3, IL-6, and TNF-α in both the brain and intestines, as well as levels of TLR4, NF-κB, LPS, and lipopolysaccharide-binding protein (LBP). Furthermore, Jiao-Tai-Wan enhances the expression of tight junction proteins (ZO-1, Occludin, and Claudin1) in intestinal tissue, advancing enhanced intestinal barrier function [89].

The flavonoids derived from *Astragalus membranaceus* have been shown to improve cognitive function in diabetic individuals by influencing the connection between the gut and the brain, enhancing mitochondrial function, maintaining the integrity of the gut barrier, and promoting the survival of neurons [90]. Key findings from *in vivo* studies reveal elevated levels of proteins and molecules associated with neuronal signaling, synaptic plasticity, and neurotransmission regulation, including postsynaptic density protein 95 (PSD95), synapsin, BDNF, and p-CREB. This suggests enhanced synaptic function and greater neuronal survival. Additionally, *Astragalus membranaceus* improves mitochondrial function by increasing the number of copies of mitochondrial DNA, PGC-1α, and p-AMPK/AMPK, as well as the expression of unfolded mitochondrial proteins LONP1, CLPP, HSP60, and HSP70, resulting in improved energy metabolism. *Astragalus membranaceus* also protects the intestinal barrier by increasing the expression of ZO-1 and claudin 5 in the hippocampus and promoting the growth of beneficial probiotics [91].

Ginseng grown under natural forest conditions (FG) has been shown to exhibit more potent anti-aging effects than garden-cultivated ginseng (GG) [92]. In old rats, FG decreased hippocampal lesions and improved weight gain while also enhancing antioxidant defenses like SOD and CAT and lowering lipid peroxidation, as seen in MDA. FG also modulated proteins involved in apoptosis (Bax, Bcl-2, Caspase 3, P53) and signaling pathways such as PI3K/AKT/mTOR, SIRT1/NF-κB, which reduced excessive cell death and inflammation in the aging brain. Treating with FG also restored diversity and balance to the gut microbiome by raising levels of good bacteria like Lactobacillus [93].

Although there is currently no cure for AD, these traditional medications have demonstrated beneficial effects on cognitive function, gut microbiota modulation, inflammation reduction, and enhancement of mitochondrial function in animal models of AD. These results offer valuable insights into potential therapeutic approaches for managing AD and emphasize more the significance of addressing Aβ protein aggregation and the gut-brain axis in creating innovative treatments for this debilitating disease.

#### 6.1.2 Parkinson's Disease (PD)

PD is a neurodegenerative disorder characterized by motor symptoms such as tremors, rigidity, and balance difficulties. Alterations in the gut microbiome (*e.g*., Bifidobacterium, Lactobacillus, and Akkermansia) may contribute to the development of the disease and pro-inflammatory status, while Faecalibacterium and Roseburia are depleted in PD patients. Inflammation can lead to the accumulation of α-synuclein, a hallmark of PD, and potentially contribute to the progression of the disease [101, 102]. While conventional drugs such as dopamine replacement and levodopa provide limited symptom relief but are associated with side effects [103], it seems herbal remedies and herbal compounds (Table **2**) are promising alternatives with fewer side effects.

Adebayo *et al*. reported that treatment with *Ginkgo biloba*, commonly known as ginkgo or maidenhair tree, improved motor function and prevented the loss of dopaminergic neurons in the brain of female mice. Additionally, this extract reduced oxidative stress by reducing MDA and Nitrite and increasing GSH, glutathione-S-Transferase (GST), catalase (CAT), and SOD and inflammation (myeloperoxidase (MPO), TNF-α, and IL-6) in the brain and gastrointestinal tract, and improved cholinergic transmission. Furthermore, this treatment was also found to increase the expression of Nrf2, reversing the damage to the ileal epithelial tissues and reducing hyperplasia of cryptal cells [104]. In line and by targeting TNF-α and ILs inflammatory mediators, an experiment on rats with induced PD was conducted. They found that a traditional Chinese medicine formula called Tianqi Pingchan Granule, given orally at a dose of 5.6 g/kg, effectively modulated the gut microbiota and inflammatory response in PD rats. Tianqi Pingchan Granule exposure led to a decrease in peripheral inflammatory cytokines, including TNF-α, IL-1, IL-2, IL-4, and IL-6, and inhibition of the activation of microglia and astrocytes in the rats' brains. Furthermore, Tianqi Pingchan Granule treatment increased the abundance of beneficial bacterial groups, Selenomonadales and Aeromonadales, in the gut microbiota [105].

In a study using 6-OHDA-induced PD rats, it was found that piperine, the primary alkaloid presents in *Piper longum*, improved autonomic movement and gastrointestinal dysfunctions, reduced the aggregation of α-Synuclein, and protected against the loss of dopaminergic neurons. Administration of piperine orally led to an increase in the ratio of light chain 3 (LC3) II/I and degradation of p62 in the substantia nigra and colon of the rats, indicating an enhancement in autophagy flux [106, 107]. Furthermore, the phosphorylation levels of PI3K, AKT, and mTOR decreased in these regions. Additionally, after this treatment, there was an increase in the levels of Bacteroides, Prevotella_CAG279, and Prevotella_ CAG1031 bacteria, while the levels of *Salmonella*, *Escherichia_coli,* and Escherichia_phage_phAPEC8 bacteria decreased. To further validate the effectiveness of piperine in reducing A53T-α-Synuclein, a mutant form of α-Synuclein associated with familial PD, experiments were conducted on A53T-α-Synuclein transgenic SH-SY5Y cells, a human neuroblastoma cell line. It was observed that piperine achieved this effect by activating the autophagy pathway mediated by PI3K/AKT/mTOR. However, this effect could be mitigated by blocking autophagy with the inhibitor bafilomycin A1 or stimulating PI3K signaling using the agonist 740 Y-P [107].

Administering chicory acid, a polyphenolic acid derived from chicory and purple coneflower, before exposure to 1-methyl-4-phenyl-1,2,3,6-Tetrahydropyridine (MPTP), a mouse model of PD, significantly decreased movement disorders [108]. This treatment also prevented the death of dopaminergic neurons in the substantia nigra, reduced glial hyperactivation, and increased neurotrophin levels in the striatum. Additionally, chicory acid partially restored the normal composition of the intestinal microbiota, specifically by decreasing the abundance of Bacteroidetes and Parabacteroide while increasing the presence of Ruminiclostridium, Lactobacillus, and Firmicutes. Moreover, chicory acid promoted the integrity of the colonic epithelium and normalized SCFA production. Interestingly, chicory acid also led to a reduction in proinflammatory cytokines like TNF-α and IL-1β in the striatum, serum, and colon, suggesting its ability to prevent neuroinflammation and intestinal inflammation. These effects may be mediated by the suppression of the TLR4/MyD88/NF-κB signaling pathway [109].

Following the administration of *Ping-wei-san* in a PD model, there was an increase in the expression of TIMP3 and NLRP6, alongside a decrease in the levels of Parkin, Gasdermin D, tissue inhibitors of metalloproteinase 3 (TIMP3), and NLRP6. The Kyoto Encyclopedia of Genes and Genomes (KEGG) pathway enrichment analysis indicated that Ping-wei-san exerts its therapeutic effects in PD through the biosynthesis of alkaloids such as ornithine, lysine, and nicotine [110, 111]. Moreover, Ping-wei-san treatment was found to reduce the levels of Lucyoside N, Arginyl-Glutamine, Niacinamide, 1,3-Disopropylbenzene, and hydroxyoctadecadienoic acids in fecal metabolites while increasing the content of VPGPR Enterostatin and dihydrowyerone acid. VPGPR is a pentapeptide classified as enterostatins, derived from procolipase, and plays a significant role in regulating fat intake, energy expenditure, and appetite. In addition, Ping-wei-san was observed to enhance the presence of Actinobacteria, Verrucomicrobiota, and firmicutes while reducing the levels of Bacteroidota, Proteobacteria, Campilobacterota, and Patescibacteria [111].

In general, these findings indicate that traditional medicine medications and associated formulations have beneficial impacts on improving motor function, modulating neuroinflammation, gut microbiota, and neuroprotective pathways, making them valuable additions to PD treatment. Additional research and clinical trials are necessary to fully understand the therapeutic benefits and mechanisms of action of these herbal medicine drugs for managing PD.

#### 6.1.3 Stroke

Stroke is a leading cause of death and disability worldwide, and its incidence is increasing in younger adults [112]. These factors can disturb the intestinal microbiota by increasing certain microbial species, such as Streptococcus, Lactobacillus, and Escherichia, and decreasing others, such as Eubacterium and Roseburia. This disruption can result in heightened inflammation, which can lead to the induction of atherosclerosis and thromboembolism, ultimately contributing to the development and progression of ischemic stroke [113, 114]. Herbal remedies (Table **3**) have been shown to have beneficial effects such as reducing inflammation, enhancing circulation, lowering blood pressure, regulating blood sugar levels, reducing cholesterol, boosting metabolism, suppressing appetite, and promoting fat burning. These effects could potentially aid in the prevention or management of stroke.

The anti-obesity properties of *Parkia biglobosa* pulp were demonstrated through its ability to control appetite, promote weight loss, and reduce fat mass in obese rats. These beneficial effects are likely attributed to the presence of bioactive compounds in the legume. These bioactive compounds have been shown to increase the populations of beneficial bacteria (such as Lactobacillus and Bifidobacterium) and decrease the populations of harmful bacteria (such as Enterobacteriaceae and Enterococcus) in the feces. Furthermore, treatment with *Parkia biglobosa* pulp resulted in a decrease in the overall number of sugars in the feces and an increase in the levels of decomposed organic acids. Moreover, this pulp exhibited antioxidant properties and modulated the oxidative parameters of lipid oxidation, antioxidant capacity, and NF-κB levels in both the intestine and brain [115].

As a major mechanism in the gut-brain axis, targeting immune neurotransmitters is of importance. In line, *Rhubarb anthraquinone* glycosides were found to decrease the levels of aspartate (Asp) and glutamate (Glu) in both the brain and colon while increasing the levels of serotonin, 5-HIAA. The administration of corn silk (*Stigma maydis*) water extract resulted in a decrease in neuronal cell death in gerbils that underwent transient cerebral ischemia and reperfusion, and it also improved neurological symptoms such as walking patterns, flexor reflex, stooping posture, and eye drooping. The neuroprotection effects of corn silk against ischemic stroke symptoms were linked to a reduction in levels of TNF-α, IL-1β, superoxide, and lipid peroxide, as well as an increase in SOD activity in the hippocampus [116]. Additionally, administration of this extract prevented post-stroke hyperglycemia by decreasing pancreatic β-cell mass and protecting against β-cell apoptosis, ultimately restoring β-cell mass. Its consumption also led to an increase in the abundance of beneficial bacteria such as Lactobacillus, Bifidobacterium, Alobaculum, and Ackermansia. Furthermore, it enhanced butyrate metabolism, as well as starch and glucose metabolism, while reducing lipopolysaccharide biosynthesis [117]. Naotaifang III is a new Chinese herbal formula used to treat ischemic stroke. It is composed of various traditional Chinese medicinal materials, including *Astragali radix, Puerariae Lobatae radix*, Di Long, Chuan Xiong, Jia, and GABA. This suggests that rhubarb anthraquinone glycosides can regulate the disrupted balance of neurotransmitters that occurs during cerebral ischemia-reperfusion injury [118]. Administering 3 doses of 11.34 g/kg of Naotaifang III by decreasing the amounts of LPS, TLR4, NF-κB, and IL-1β and impacting the LPS/TLR4 signaling pathway in the microbiota-gut-brain axis exerted a protective effect against neuroinflammatory injury following middle cerebral artery occlusion (MCAO) [119]. In a similar MCAO rat model, pre-treatment with raw rhubarb significantly reduced the size of the cerebral infarct area and alleviated inflammation (TNF-α, ILs). Furthermore, Raw rhubarb demonstrated the ability to regulate the integrity and permeability of the intestinal barrier, effectively countering gut microbiota dysbiosis caused by ischemic stroke, particularly the increased levels of Firmicutes. Notably, utilizing pseudo-GF rats indicated that the positive impact of Raw rhubarb on stroke outcomes may be reliant on the presence of a healthy intestinal microbiota [120].

*Eleutherococcus senticosus* (Rupr. & Maxim.) Maxim, also known as Siberian ginseng, has been discovered to alleviate the imbalance of intestinal microbiota caused by ischemic stroke *via* modulated bile acids such as cholic acid, chenodeoxycholic acid, tauroursodeoxycholic acid, ursodeoxycholic acid, deoxycholic acid, glycochenodeoxycholic acid, glylilithocholic acid, lithocholic acid, taurocholic acid, taurodeoxycholic acid, and taurolithocholic acid and reducing the pro-inflammatory cytokines IL-17, IL-1α, IL-1β, COX-2 and TNF-α [121]. Also, this treatment led to changes in the level of neurotransmitters (5-HT, dopamine, epinephrine, GABA, glutamate, and norepinephrine). Also, after treatment with this extract, the levels of Proteobacteria, *Escherichia-Shigella*, Enterobacter, and Oscillibacter bacteria decreased [122].

By targeting metabolic pathways, *Dengzhan shengmai*, a traditional Chinese medicine formula consisting of Erigeron breviscapus, Schisandra chinensis, Panax ginseng, and Ophiopogon japonicus, has been studied by Guo *et al*. for its potential in ameliorating cerebral ischemia through the gut-brain axis. It was suggested that SCFAs play a crucial role in mediating the effects of *dengzhan shengmai*. The increased levels of SCFAs resulted in the upregulation of monocarboxylate transporters (MCT), which inhibited neurocyte apoptosis *via* the PI3K/AKT/Caspase-3 pathway [123, 124]. Furthermore, the elevated levels of SCFAs in the intestines contributed to the restoration of gut barrier integrity and prevented the translocation of LPS from the intestine to the brain, thereby reducing neuroinflammation (TNF-α, IL-1β, and IL-6) and improving cognition-related dysfunction in 2VO model. This effects was associated with increase in levels of Prevotellaceae, Bacteroidaceae, Alloprevotella, Bacteroidetes, Lactobacillus, Allobaculum, *Ruminococcus torques, Ruminococcus gauvreauii* and decrease in Anaerostipes, Monoglobaceae, Monoglobus, Erysipelotrichaceae, Erysipelatoclostridium, Oscillospira, Lachnospiraceae_ND3007, Lachnospiraceae_UCG-004, Candidatus Saccharimonas [124].

By targeting the neuroimmune system of the gut-brain axis, Pang *et al*. showed that consuming 15 mL/kg of *Dioscorea polystachya*, also known as “yam gruel” or Chinese yam, daily for 4 weeks could enhance cognitive function in diabetic rats with cerebral ischemia-reperfusion injury [125]. This improvement was accompanied by reductions in the levels of LPS, MDA, TNF-α, IL-1β, and fasting blood glucose. Additionally, there were increases in the production of SCFAs (Acetic acid, Propionic acid, Butyric acid) in both the colon and cerebral cortex, as well as elevated levels of SOD, GABA, 5-HT, and BDNF [126]. Furthermore, the treatment with “yam gruel” increased the presence of beneficial bacteria such as Lactobacillus, Ruminococcus, and Clostridium while decreasing the ratio of Firmicutes to Bacteroidetes [125].

Overall, herbal remedies show potential as effective strategies for preventing and managing strokes by reducing inflammation, regulating metabolism, affecting neurotransmitter activity, and impacting the gut-brain connection.

## 7 INSIGHTS INTO CLINICAL TRIALS ON THE GUT-BRAIN AXIS AND FUTURE PROSPECTS

The human gut microbiome is highly responsive to dietary changes, with significant alterations occurring rapidly upon the introduction of different diets. A pivotal study published by David *et al*. demonstrated that short-term consumption of diets composed entirely of animal or plant products can dramatically change the microbial community structure in the gut within just a few days. This research highlights how dietary composition can overwhelm individual differences in microbial gene expression, indicating a strong link between diet and microbiome dynamics. The study found that an animal-based diet increased the abundance of bile-tolerant microorganisms such as Alistipes, Bilophila, and Bacteroides while decreasing levels of Firmicutes that are involved in metabolizing plant polysaccharides, such as Roseburia, *Eubacterium rectale*, and *Ruminococcus bromii* [127].

Conventional Chinese medications such as *Triphala*, *Danggui-Shaoyao-San*, and ZiBuPiYin Formula have appeared to guarantee reestablishing gut-brain work and giving neuroprotection by tweaking the intestine microbiome and smothering fiery pathways in creature models [128]. The synthetic copolymer glatiramer acetate, FDA-approved for treating MS, has shown potential for neuroprotection and cognitive preservation in several neurological conditions involving cognitive decline, based on clinical studies and animal models [129]. Natural compounds and dietary supplements have shown neuroprotective effects in experimental and clinical glaucoma, an optic neuropathy characterized by the progressive death of retinal ganglion cells. This provides alternative strategies for disease management that are independent of intraocular pressure [130].

In a clinical trial conducted over 5 weeks and in 4 groups of 92 healthy individuals with mild to moderate levels of anxiety and depression, the effects of oligofructose, 2’fucosyllactose alone, and the combination were compared with maltodextrin, focusing on their impact on the gut-brain axis. The results showed a significant increase in beneficial bacterial species such as Bifidobacterium, Bacteroides, Roseburia, *Faecalibacterium prausnitzii*, and Prevotella when combined oligofructose and oligofructose/2’fucosyllactose were used. These interventions demonstrated better performance in improving the mood of individuals through their prebiotic effects on the gut-brain axis [131]. In another study, inulin extracted from herbal medicine sources was used as a prebiotic alongside Bifidobacterium animalis subsp. Lactis GCL2505 led to a significant improvement in cognitive scores of healthy people who had mild cognitive decline due to aging. This study indicated that inulin increased the number of bacteria in the stool, potentially leading to cognitive improvement through this mechanism [132].

Since evidence suggests that polyphenol-rich compounds may significantly alter gut microbiota diversity [133], polyphenols present in extra virgin olive oil can have significant effects on the gut-brain axis. A clinical trial was conducted to examine these effects in healthy adults through the gut-brain axis. The study will involve thirty healthy adult participants aged 18 to 35. Each participant will consume a daily dose of 0.7 g of Corbella extra virgin olive oil/kg of body weight alongside their regular diet (NCT05898113). Another clinical trial will assess the effects of polyphenols in cranberries through the gut-brain axis on mood disorders underway for 12 weeks (NCT05260346). In the phase 2 clinical trial, the effects of polyphenols liposomal alongside probiotic G04CB02 were studied in improving ALS patients by influencing gut bacteria for 4 months (NCT04654689). In a clinical trial, the daily consumption of 30 g of dark chocolate (85% and 70%)-extractable from cocoa beans- for 3 weeks on the mood of healthy individuals, a significant reduction was observed in negative symptoms in chocolate consumers. Analysis of gut microbiota showed an increase in Blautia obeum and a decrease in *Faecalibacterium prausnitzii* in consumers of 85% dark chocolate. These results indicated that dark chocolate, with its prebiotic effect, alters gut microbiota and improves mood through its impact on the gut-brain axis [134].

Polydextrose, a non-digestible oligosaccharide, was beneficial in improving cognitive function and acute stress responses by altering gut microbiota and subsequently affecting the gut-brain axis. In a clinical trial involving 18 healthy female participants, subjects received either 12.5 g of Litesse^®^ Ultra (comprising over 90% polydextrose) or maltodextrin for a duration of 4 weeks, although gut bacterial diversity was not changed, the abundance of beneficial bacteria Ruminiclostridium increased compared to the placebo group [135]. Tomatoes and tomato-based products, and other products containing lycopene have phytochemical metabolites that can directly act as neurotransmitters in the CNS or indirectly activate and alter the gut-brain axis. A clinical trial currently underway is investigating the daily consumption effects of these products over three months (NCT059891977). In 2023, a clinical trial will examine the effects of yeast beta-glucan on patients with mild cognitive impairment through its impact on gut microbiota (NCT06083350). In another clinical trial of 2023, the effects of caffeine as a prebiotic on improving mood, memory, and cognitive function through the gut-brain axis will be studied in a clinical trial over 3 weeks (NCT05927038).

A retrospective analysis of 39 medical records from PD patients with chronic constipation who received a probiotic supplement (comprising butyrate triglyceride 302.86 mg, *Crocus sativus* L. or saffron 30 mg, and vitamin D3 100 mcg) demonstrated a significant improvement in median defecation frequency, along with a 7.7% reduction in the Unified Parkinson’s Disease Rating Scale III (UPDRS III) score. These findings suggested that addressing alterations in the gut microbiome through probiotic supplementation may provide potential relief from both motor and non-motor symptoms associated with PD [136]. A double-blind, parallel randomized controlled trial involving 61 healthy older adults examined the effects of daily consumption of 26 g of wild blueberry (poly)phenols, which contains 302 mg of anthocyanins, compared to a placebo over a 12-week period. The results demonstrated significant improvements in endothelial function (0.86%) and reductions in 24-hour ambulatory systolic blood pressure (-3.59 mmHg) in the wild blueberry group, as well as enhanced cognitive performance. However, no significant changes were observed in cerebral blood flow or gut microbiota composition [137]. The effects of Cereboost^®^, an American ginseng extract, on cognition and mood were assessed in three phases: acute (first 6 hours post-intervention), chronic (after 2 weeks of daily supplementation), and acute-on-chronic (response to a single dose after chronic use) in a double-blind, randomized, placebo-controlled trial with 61 participants. Results showed that Cereboost^®^ improved working memory and attention immediately, with effects amplified after two weeks. Chronic use also enhanced performance on acetylcholine-sensitive tasks and improved mood by reducing mental fatigue and increasing self-assurance [138]. The results of a randomized, controlled, cross-over trial involving 66 participants aged 60 and older revealed that after an 8-week polyphenol-rich diet, serum zonulin levels significantly decreased, indicating reduced intestinal permeability. Furthermore, the polyphenol-rich diet was linked to lower diastolic and systolic blood pressure, especially among participants not taking antihypertensive medications. Additionally, there was a notable increase in beneficial gut bacteria, specifically fiber-fermenting and butyrate-producing species such as those from the family Ruminococcaceae and the genus Faecalibacterium [139].

These results underscore the potential of conventional medications and natural substances in safeguarding neurons. Nevertheless, additional research is necessary to elucidate the precise mechanisms, refine dosing protocols, and apply these encouraging findings in a clinical setting. Also, extensive clinical trials are required to validate the effectiveness, safety, and lasting effects.

Altogether, there is a growing interest in highlighting the role of gut microbiota in neurodegenerative conditions and the involvement of multiple key players in the gut-brain axis. The axis includes vague nerve, immune pathway, HPA, and metabolic pathways, thereby regulating several inflammatory pathways in the nervous system [140]. Of major central mechanisms, immune dysregulation, BBB permeability, microglia activation, and protein aggregation play major roles in the inflammatory aspects of neurodegeneration. The impact of medicinal herbs on gut microbiome and interconnected gut-brain mediators leads to the regulation of neuronal factors [141], with differential effects on mitochondria and also apoptotic/oxidative pathways [142, 143]. This allows medicinal herbs to regulate gut-microbiota, gut-brain axis, and interconnected signaling mediators.

## CONCLUSION

**Fig. (3):**
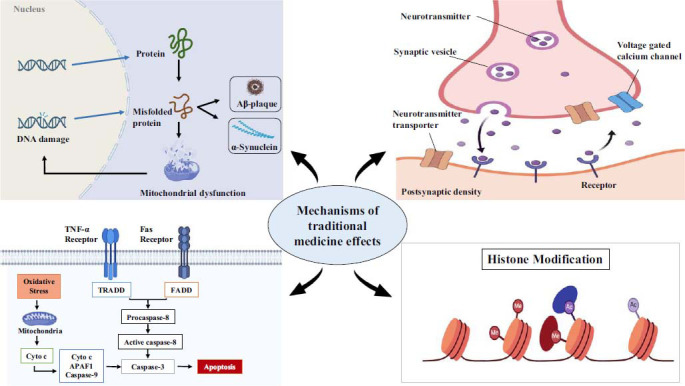
Anti-inflammatory effects of traditional medicine mediated by the gut-brain axis. Created with www.biorender.com.

The intricate interplay between brain and gut microbiota is increasingly recognized as a critical factor in the development and advancement of NDDs, with inflammation serving as a pivotal player in this relationship. By leveraging the anti-inflammatory properties of medicinal herbs on the gut microbiota, significant therapeutic benefits have been observed in various conditions such as mood disorders, AD, PD, and stroke. Accordingly, medicinal herbs promote the growth of beneficial bacteria and reduce harmful ones to attenuate inflammatory agents (*e.g*., microglia, protein aggregation, ILs, TNF‐α, NF-κB, COX-2, and TLR-4), signaling pathways (*e.g*., metabolic pathway, neurologic pathway, HPA, immune dysregulation) and related signaling mediators (*e.g*., PI3K/Akt/mTOR and ERK/MAPK) (Fig. **3**).

Since pharmacokinetics plays a crucial role in drug discovery and development, focusing on how drugs are absorbed, distributed, metabolized, and eliminated (ADME) by the body and considering the pharmacokinetic limitations of herbal medicine, employing novel delivery systems (*e.g*., nanoparticles and microcapsules) would increase the bioavailability and efficacy of herbal medicine. These novel delivery systems can also facilitate targeted delivery to the gut and allow for more precise modulation of gut microbiota and inflammation.

The current systematic and comprehensive review highlighted the potential of multi-targeting medicinal herbs in restoring gut-brain function and promoting neuroprotection through targeting inflammatory pathways. Looking ahead, further research is imperative to validate the efficacy and safety of medicinal herbs through well-designed clinical trials.

Of limitations, since there are complex pathophysiological mechanisms behind neurodegenerative diseases, the current study focused on the neuroinflammatory aspects targeted by medicinal herbs.

More reports could also focus on other apoptotic, oxidative stress and autophagy-related pathways.

## Figures and Tables

**Fig. (2):**
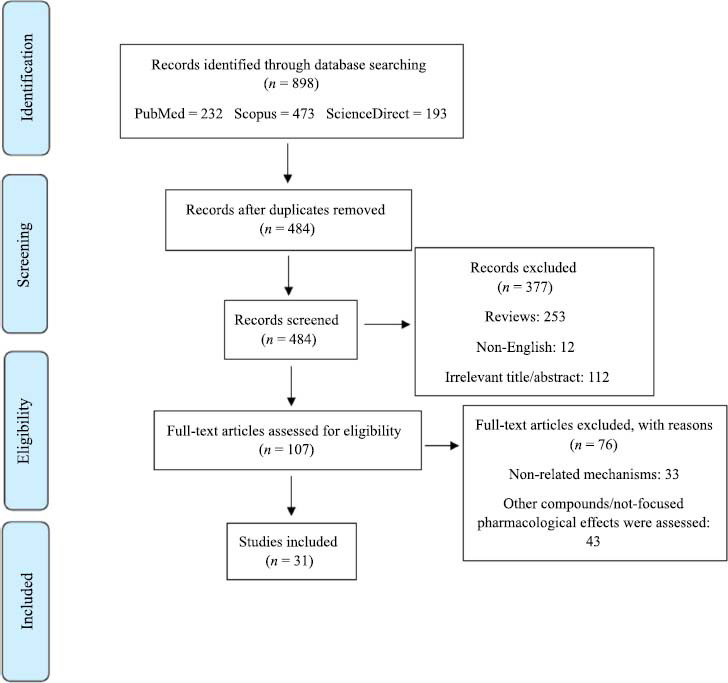
The PRISMA flowchart on the literature search procedure and selection of related studies.

**Table 1:** Effects of traditional medicine on the gut microbiota in Alzheimer's diseases.

**Traditional Medicine**	**Disease**	**Animal/Dose**	**Model**	**Effects**	**Active Chemical Compounds/Source/ Extraction**	**Plant Origin**
Korean Red Ginseng [78]	AD	Male Tg2576 heterozygous transgenic mice 30, 100 mg/kg	Tg2576 heterozygous transgenic mice express the human APP	↑ Lactobacillus ↓ Bifidobacterium, Ruminococcus, Clostridia, Anaerostipes ↑ Occludin, Claudin-5, Laminin ↓ Aβ, Iba-1	Triterpene saponins (Ginsenosides)/Korea/ Water extract	Root
*Triphala* [79-80]	AD	C57BL/6 and 5xFAD, APP/PS1 mice 500 mg/kg 60 days	The 5xFAD and APP/ PS1 mice express human APP	↑ Bacteriodetes, Verrucomicrobiota, Akkermanasia, Lactobacillus, ↓ Deferribacteres, TM7, Proteobacteria, Blautia, Helicobacter, Alistipes, Oscillospira, Desulfovibrio ↑ 5-HT, Ach, BDNF ↓ TNF-α, IL-6, IL-1β, IL-10, IL-17, IFN, TLR4, SOD1, Aβ, APP, BACE, NPY, TREM2, LPO, LPS, Blood glucose	N.R./India/Ethanol extract	Fruit of *Emblica officinalis*, Fruit of *Terminalia chebula* and *Terminalia belerica*
*Forsythiae Fructus* and *Cassiae Semen* [81]	AD	Male SD rats 200 mg/kg 8 weeks	Aβ_25-35_	↑ Bacteriodales ↓ Clostridales, Desulfovibrionales, Enterobacteriales ↑ TNF-α, IL-1β, MDA, BDNF, CNTF, Glycogen ↓ Akt, GSK-3β, FOXO1, tau, Triglyceride	Phenolic compounds/ Korea/Water extract	Fruit Seed
Coptis chinensis Franch [82]	AD	SPF male C57BL/6 mice SPF male APPswe/PS1ΔE9 mice 0.30, 0.75 g/kg 6 weeks	APPswe/ PS1ΔE9	↓ Linoleic acid-biotin, 4-Indolecarbaldehyde, N-Acetylneuraminic acid, Eicosapentaenoic acid, Hydrolyzed fumonisin B1, Adenine, Guanine, Stearamide, 2′-Deoxyadenosine, Methyl isonicotinato ↑ Acetic acid, Indole-3-lactic	Alkaloids (Isoquinoline)/ China/ Ethanol extract	Rhizome
*Codium fragile* [83]	Cognitive impairment	Male BALB/c mice 50, 100 mg/kg 12 weeks	Particulate matter	↑ Firmicutes, Lachnospiraceae, Oscillospiraceae, Rikenellaceae, Alistipes, Lachnospiraceae NK4A136 group, Desulfovibrio, Colidextribacter, Oscillibacter ↓ Bacteroides, Bacteroidaceae, Prevotellaceae, Muribaculaceae, Clostridia_UCG-014, Muribaculum ↑ Pro- Caspase-3, MMP, GSH, SOD, Acetate, Propionate, Claudin-1, Occludin, ACh, AChE ↓ TNF-α IL-1β, TLR-4, MyD88, p-JNK, p-NF-κB, p-IκB-α, COX-2 Caspase-1, Bax/Bcl-2, ROS MDA, MPO	Fatty acids (Stearidonic acid, Linolenic acid, Palmitoleamide, Linoleamide, Hexadecanamide, Oleamide, Octadecanamide)/Korea/Water extract	Green alga
Danggui Shaoyao San [85]	AD	Male SD rats 24 g/kg 11 days	Streptozotocin	↑ Ligilactobacillus, Muribaculaceae_unclassified, ↓Firmicutes_unclassified, Dubosiella, Bifidobacterium ↑ SOD, CAT, GSH-PX ↓ TNF-α, IL-1β, IL-6, MDA	Phenolic acids (Gallic acid, Ferulic acid, Benzoic acid, Coniferyl Ferulate) Monoterpene Glycosides (Paeoniflorin, Albiflorin) Phthalides (Z-Ligustilide, Senkyunolide A, Z-butylidenphthalide, 3-butylphthalide, Senkyunolide I, Levistolide A) Sesquiterpenoids (Atractylcnolide II, Atractylcnolide I) Triterpenes/china/Water extract	Root of *Angelica sinensis*, The root of *Paeonia lactiflora*, Rhizome of *Ligusticum chuanxiong*, Rhizome of *Alisma orientalis*, Sclerotium of *Poria cocos*, Rhizome of *Atractylodes macrocephala*
*Rhizoma gastrodiae* [86]	Cognitive impairment	Mice 100, 200, 300 mg/kg 8 weeks	D-galactose/ Aluminum chloride	↑ Firmicutes, Bacilli, *Lactobacillus johnsonii, Lactobacillus murinus, Lactobacillus reuteri*	Phenolic glucosides (Gastrodin)/China/ Water extract	Rhizome
*Eucommia ulmoides* [87]	Cognitive impairment	Male C57BL/6 mice 100, 200, 400 mg/kg 3 weeks	Dextran sulfate sodium	↑ MMP, GSH, SOD, p-AKT, MUC2, ZO-1, Occludin, Claudin-1, Acetic acid, Butyric acid, ATP, ACh, ChAT, ↓ TNF-α, IL-1β, My88, TLR-4, MyD88, p-NF-κB, p-IκB-α, COX-2, MDA, ROS, iNOS, Caspase-1, Caspase-3, Bax/Bcl-2, p-JNK, MPO, Albumin, AChE	Polyphenol (Chlorogenic acid) Phenolic compound (Caffeic acid) Flavonoid (Quercetin) Monoterpenoid (Aucubin)/Korea, China, Japan/Methanol extract	Leaves
*Acanthopanax senticosus* [88]	Cognitive impairment	Male KM mice 250 mg/kg	^60^Co-γ ray irradiation	↑ Lactobacillus ↓ Helicobacter ↑ IκBα, BDNF, 5-HT, ACH, NE ↓ TNF-α, IL-6, IL-1β, NF-κB, GABA	Phenolic compound (Protocatechuic acid, Isofraxidin, Chlorogenic acid, Eleutheroside E)/China/ Methanol extract	Root, Stem
Jiao-Tai-Wan [89]	Cognitive impairment	Male SD rats 1.1, 2.2 g/kg 8 weeks	Obesity-resistant rats with partially sleep-deprived	↑ ZO-1, Occludin, Claudin1. ↓ TNF-α, IL-6, TLR4, NF-κB, TLR4, MyD88, p-IKKβ/ IKKβ, p-P65, P65, Cspase3, Aβ42, LPS, LBP,	Alkaloid (Coptisine hydrochloride, Palmatine chloride, Berberine hydrochloride, Jatrorrhizine hydrochloride), Flavonoid (Cinnamaldehyde) Cinnamon acid, / China/Water extract	Rhizome of Coptidis, Cortex of Cinnamomi
*Astragalus membranaceus* [91]	Cognitive impairment	Male C57BL/6J mice 50, 25, 5 mg/kg 16 weeks	Streptozotocin	↑ p_Acidobacteria, p_Latescibacteria, p_Gemmatimonadetes, p_Proteobacteria, p_unidentified_Bacteria, p_Deferribacteres, g_Faecalibacterium, g_Bifidobacterium, g_Aerococcus ↑ p_Tenericutes, p_Bacteroidetes, p_Melainabacteria, p_Chloroflexi ↑ BDNF, p-CREB/CREB, PSD95, Synapsin, p-AMPK/ AMPK, PGC-1α, LONP1, CLpP, ZO-1, Claudin 5, 5-HT, HSP60, HSP70 ↓ TNF-α, IL-6, IL-1β, GABA, LPS, Aβ	Isoflavonoid (Calycosin, Ononin, Calycosin-7-O-beta-d-glucoside, Formononetin)/ China/N.R.	Rhizoma of *Coptis chinensis*, Bark of Cinnamomum cassia
Ginseng [93]	Cognitive impairment	Mice 400 mg/kg 6 weeks	D-galactose	↑ Lactobacillus ↑ SOD, CAT, Bcl-2, p-AKT, SIRT1, ↓ MDA, NF-κB, Caspase 3, Bax, P53, p-PI3K, mTOR	Triterpene saponins (Ginsenosides), polyphenols, crude polysaccharides, starch, Protein/ China/ Water extract	Root
*Rhodiola rosea* [94]	AD	Male SAMR1 and SAMP8 mice, 50 mg/ kg 13 weeks	Aβ_1–42_	↑ Clostridiales_vadinBB60, Streptococcaceae, norank_f_Clostridiales_, vadinBB60, Peptococcus, Streptococcus, Ruminococcaceae_UCG_009 ↓ TNF-α, IL-6, IL-1α, IL-17A, IL-12, IL-1β, Aβ	Phenylpropanoid glycoside (Salidroside)/ China/N.R.	Root
*Xanthoceras sorbifolia Bunge* [95]	AD	Male SD rats 0.056, 0.112, 0.224 mg/kg	Aβ_1–42_	↑ Methanomassiliicoccus, Azoarcus, Phycisphaera, Acetobacteroides, Alloprevotella ↓ Firmicutes/Bacteroidetes, Clostridium IV, Enterorhabdus, Coriobacterium, Corynebacterium, Desulfovibrio, Defluviitalea	Triterpenoid Xanthoceraside/China/ Ethanol extract	Husk
Kai-Xin-San [96]	AD	SPF-grade male SD rats 10 g/kg 6 Weeks	Aβ_25-35_	↑ Lactobacillus, Limosillactobacillius, Blautia Ligilactobacillus, Alloprevotella, Prevotellaceae_NK3B31_group, Alistipes ↓ Firmicutes/Bacteroidetes, Allobaculum, Clostridium_sensu_stricto_1 ↓ TNF-α, IL-1β, IL-6	Phenylpropanoid (α-asarone and β-asarone) Triterpene saponins (Ginsenosides)/ Oligosaccharide ester (3,6′-disinapoyl sucrose) Triterpenoid (Pachymic acid)/China/Ethanol extract	Root and rhizome of *Panax ginseng*, Root of *Polygala tenuifolia*, Rhizome of *Acorus tatarinowii,* Sclerotium of *Poria cocos*
Danggui Shaoyao San [84]	Cognitive impairment	Male C57BL/6N mice 4.8 g/kg 2 weeks	Scopolamine	↑ Bacteroides/Firmicutes, Proteobacteria, ↓ Verrucomicrobia ↑ PPAR-γ, ZO-1, Occludin, HDL-C, APDN, LXR, ↓ TNF-α, IL-6, MDA, TG, LDL-C, T-CHO	Phenolic acids (Gallic acid, Ferulic acid, Benzoic acid, Coniferyl Ferulate) Monoterpene Glycosides (Paeoniflorin, Albiflorin) Phthalides (Z-Ligustilide, Senkyunolide A, Z-butylidenphthalide, 3-butylphthalide, Senkyunolide I, Levistolide A) Sesquiterpenoids (Atractylcnolide II, Atractylcnolide I) Triterpenes/China/N.R.	Root of *Angelica sinensis*, The root of *Paeonia lactiflora*, Rhizome of *Ligusticum chuanxiong*, Rhizome of *Alisma orientalis*, Sclerotium of *Poria cocos*, Rhizome of *Atractylodes macrocephala*
Dangshen Yuanzhi [97]	Memory disorder	SPF Male and Female SD rats 6.67, 13.34 g/kg 5 weeks	D-galactose/ Aluminum chloride	↓ MCP-1, NF-L, NSE ↑ Bacteriodales, Prevotella ↓ Firmicutes, Lactobacillus	N.R./ China/Ethanol extract	Root
Chaihu Shugan San [98]	AD	Male SAMP8 and SAMR1 mice 2.1 g/kg, 4.2 g/kg 8 weeks	Sporadic AD	↑ *Lactobacillus reuteri* ↓ *Staphylococcus xylosus*	N.R./China/ Water extract	Root of *Bupleurum chinense*, Tuber of *Cyperus rotundus,* Fruit peel of *Citrus reticulate*, Fruit peel of *Citrus* × *aurantium*, Rhizome of *Ligusticum chuanxiong*, Root of *Paeonia lactiflora*, Root of *Glycyrrhiza uralensis*
beans and pulses [99]	Cognitive impairment	C57BL/6J mice 200 μL per dose; 425 g/L 20 weeks	Aging	↑ Bacteroides, Parabacteroides, Alistipes, Bilophila, Butyricimonas, Akkermansia ↓ Streptococcus ↑ IL-10, Claudin-1, Claudin-5, JAM3, ZO-1, Occludin, Butyrat ↓ TNF-α, IL-1β, IL6, IL8, IFNɣ, p16, p21, ALT, AST, Leptin, Insulin, Leucine	Resistant starches/ N.R./N.R.	Seed
ZiBuPiYin [100]	Cognitive impairment	Male Zucker diabetic fatty rats, Lean Zucker rat 34.6 g/kg, 17.3 g/kg, 8.7 g/kg 10 weeks	Zucker diabetic fatty	↑ Parabacteroides, Bacteroides, Butyricimonas, Prevotella, Lactobacillus, Ruminococcus ↑ HbA1c, RBG, HOMA-IR, FSI, p-AKT, L-aspartic acid, L-lysine, L-isoleucine, L-leucine, L-glutamic acid, Butyric acid, Valeric acid, Acetic acid, Propionic acid, Isobutyric acid ↓ p-IRS2, FOXO1	N.R./China/Water extract	Root of *Red Ginseng*, Rhizome of Common Yam, The root of Poria, The root of White Peony, Root of Red Sage, Bean of Hyacinth Bean, Seed of Lotus Seed, Rhizome of Grass-Leaved Sweetflag, Root of Thinleaf Milkwort, Wood of Sandalwood, Epicarp of Tangerine Red Epicarp, Root of Licorice

**Abbreviations**: 5‐hydroxytryptamine (5‐HT), Alzheimer's disease (AD), Acetylcholine (ACh), Adenosine triphosphate (ATP), Adiponectin (APDN), Amyloid precursor Protein (APP), Brain-derived neurotropic factor (BDNF), Beta secretase (BACE), Catalase (CAT), Choline acetyltransferase (ChAT), Ciliary neurotrophic factor (CNTF), c-Jun N-terminal kinases (JNKs),), Cyclooxygenase 2 (COX-2), Fasting serum insulin (FSI), Glutathione (GSH), Glycogen synthase kinase-3 beta (GSK-3β), Glycosylated hemoglobin (HbA1), Heme oxygenase-1 (HO-1), High-density lipoprotein cholesterol (HDL-C), Homeostasis model of assessment for insulin resistance (HOMA-IR), Inducible nitric oxide synthase (iNOS), Interleukin (IL), Low-density lipoprotein cholesterol (LDL-C), LPS binding protein (LBP), Malondialdehyde (MDA), mitochondrial membrane potential (MMP), Myeloid differentiation primary response protein 88 (MyD88), Myeloperoxidase (MPO), Nuclear factor κ-light-chain-enhancer of activated B cells (NF-κB), Neurofilament light chain protein (NF-L), Neuron-specific enolase (NSE), Not reported (N.R.), Peroxisome proliferator activated receptor gamma (PPAR-γ), Phosphoinositide 3-kinases (PI3Ks), postsynaptic density protein 95 (PSD95), Protein kinase B (AKT), Random blood glucose (RBG), Silent information regulator 1 (SIRT1), Superoxide dismutase (SOD), Specific-pathogen free (SPF), Toll-like receptor 4 (TLR4), Total glyceride (TG), Tumor necrosis factor‐α (TNF‐α), Zonula occludens-1 (ZO-1), γ‐aminobutyric acid (GABA).

**Table 2:** Effects of traditional medicine on the gut microbiota in Parkinson's disease.

**Traditional Medicine**	**Disease**	**Animal/Dose**	**Model**	**Effects**	**Active Chemical Compounds/Source/ Extraction**	**Plant Origin**
*Ginkgo biloba* [104]	PD	Male Swiss mice 20 mg/kg 10 days	Rotenone	↑ GSH, GST, CAT, SOD ↓ TNF-α, IL-6, Nrf2, Caspase-3, MDA, Nitrite, AChE, MPO	Flavonoids Terpene lactones (Ginkgolides B, Bilobalide) Phenolic compounds (Ginkgolic acids)/USA/N.R.	Leave
*Tianqi pingchan granule* [105]	PD	Male SD rats 5.6 g/kg	6-hydroxy-dopamine	↑ Selenomonadales, Aeromonadales ↓ TNF-α, IL-6, IL-1, IL-2, IL-4, GFAP	N.R./China/N.R.	Fruit of *Lycium barbarum* L., The whole plant of *Taxillus chinensis* (DC.) Danser, Tuber of Gastrodia elata Blume, The root of *Paeonia lactiflora Pall*., Tuber of *Arisaema erubescens* (Wall.) Schott, Rhizome of *Curcuma phaeocaulis* Valeton
*Piper longum* [107]	PD	Male SD rats 10, 20, 40 mg/kg 5 weeks SH-SY5Y cell 10, 20 40 μM	6-hydroxy-dopamine A53T-α- Synuclein	↑ Bacteroides, Prevotella_CAG2, Prevotella_ CAG1031 ↓ Salmonella, *Escherichia_coli*, Escherichia_phage_ phAPEC8 ↑ LC3II/LC3I, Acetic acid, Propanoic acid, Bbutyric acid, ↓ p62, p-PI3K, p-AKT, p-mTOR, Tyrosine hydroxylase, α-Synuclein	Alkaloid (Piperine)/ China/N.R.	Leave
Chicory, Purple coneflower (*Echinacea purpurea*) [109]	PD	Male C57BL/6J mice 30, 60 mg/kg 12 days	MPTP	↑ Firmicutes, Lactobacillus, Ruminiclostridium. ↓ Bacteroidetes, Parabacteroide ↑ ZO-1, Occludin, BDNF, GDNF, Tyrosine hydroxylase, ↓ TNF-α, IL-1β, TLR4, MyD88, NF-κB, Acetic acid, Propionic acid, Isobutyric acid, Butyric acid, Isovaleric acid, Valeric acid	Phenolic acids (Chicory acid)/China/N.R.	Leaves of the chicory plant, Aerial parts of the purple coneflower
Ping-wei-san [111]	PD	Female C57BL/6 mice 975 mg/kg 30 days	-	↑ Actinobacteria, Verrucomicrobiota ↓ Bacteroidota, Proteobacteria, Campilobacterota, Patescibacteria ↓ Parkin, GSDMD, TIMP3, NLRP6	N.R./China/N.R.	Rhizome of Atractylodis, *Magnolia officinalis*, *Pericarpium Citri Reticulata, Anemarrhena asphodeloides Bunge, Rhizoma Coptidis, Aucklandia lappa* DC., Cistanche deserticola Y.C. Ma, *Radix glycyrrhizae,* components are classified

**Abbreviations**: 1-methyl-4-phenyl-1,2,3,6-tetrahydropyridine (MPTP), Acetylcholinesterase (AChE), Brain-derived neurotropic factor (BDNF), Catalase (CAT), Gasdermin D (GSDMD), Glial fibrillary acidic protein (GFAP), Glial cell line-derived neurotrophic factor (GDNF), Glutathione (GSH), Glutathione-S-Transferase (GST), Interleukin (IL), Light chain 3 (LC3), Malondialdehyde (MDA), Myeloid differentiation primary response protein 88 (MyD88), Myeloperoxidase (MPO), Mammalian target of rapamycin (mTOR) Nuclear factor erythroid 2-related factor 2 (Nrf2), Nuclear factor κ-light-chain-enhancer of activated B cells (NF-κB), Nucleotide-binding oligomerization domain, leucine rich repeat and pyrin domain containing proteins-6 (NLRP6), Not reported (N.R.), Phosphoinositide 3-kinases (PI3Ks), Protein kinase B (AKT), Parkinson's disease (PD), Silent information regulator 1 (SIRT1), Superoxide dismutase (SOD), Tissue inhibitors of metalloproteinase 3 (TIMP3), Toll-like receptor 4 (TLR4), Tumor necrosis factor‐α (TNF‐α), Zonula occludens-1 (ZO-1).

**Table 3:** Effects of traditional medicine on the gut microbiota in stroke.

**Traditional Medicine**	**Disease**	**Animal/Dose**	**Model**	**Effects**	**Active Chemical Compounds/Source/ Extraction**	**Plant Origin**
Corn silk (Stigma maydis) [117]	Ischemic stroke	Male Mongolian gerbils 0.05%, 0.2%	TFI/R	↑ Lactobacillus, Bifidobacterium, Allobaculum, Akkermansia ↓ Clostridium, *Desulfovibrio, Oscillospira* ↑ SOD, Acetate, Butanoate ↓ TNF-α, IL-1β, MDA, LPS, Insulin, Glucose, HDL, Triglyceride, NBT	Flavonoids (Luteolin 7-O-Neohesperidoside, 2″-O-α-L-Rhamnosyl-6-C-fucosyl-3′-methoxyluteolin, 2″-O-α-L-rhamnosyl-6-C-quinovosylluteolin, 2″-O-α-L-rhamnosyl-6-C-fucosylluteolin, Maysin-glycoside, 3′-methoxymaysin), Terpenoids (cis-α-terpinol, Citronellol, 6,11-oxidoacor-4-ene, Trans-pinocamphone, Eugenol, neo-iso-3-thujanol, and cis-sabinene hydrate)/ N.R/N.R.	Corn
Rhubarb anthraquinone glycosides [118]	Ischemic stroke	Male SD rats 7.5, 15, 30 mg/kg 1 week	MCAO/R	↑ 5-HT, 5-HIAA, GABA ↓ Glu, Asp	phenolic compounds (Anthraquinone glycosides)/China/Methanolic extract	Stalk
Naotaifang III [119]	Ischemic stroke	Male SD rats 11.34 g/kg 3 days	MCAO	↓ Bacteroidetes ↓ IL-1β, TLR4, NF-κB, LPS	Isoflavone (Puerarin) Phenolic compound (Ferulic Acid, Emodin) Triterpene (Astragaloside IV) /China/ Water extract	Root of *Astragalus membranaceus*, Root of *Pueraria lobate*, Rhizome of *Ligusticum chuanxiong*, Root of *Scutellaria baicalensis*, Root and rhizome of *Rheum palmatum*, Whole plant of *Taraxacum mongolicum*, Rhizome of *Zingiber officinale*, Root and rhizome of *Glycyrrhiza uralensis*
Rhubarb [120]	Ischemic stroke	Male SD rats 360, 540, 810 mg/kg	MCAO/R	↑ Firmicutes ↓ Proteobacteria, Actinobacteria, *Escherichia-Shigella* ↑ Claudin-5, ZO-1, Occludin, Claudin 5 ↓ TNF-α, IL-1β, IL-6, MMP9, D-lactate, LBP	N.R./China/Raw powder	Stalk
*Eleutherococcus senticosus* (Siberian ginseng) [122]	Ischemic stroke	Male SD rats 100 mg/kg 4 weeks	Two-vessel occlusion	↓ Proteobacteria, Escherichia-Shigella, Enterobacter, Oscillibacter ↑ IL-10, SOD, Glycine, Cholic acid, Chenodeoxycholic acid, Tauroursodeoxycholic acid, Ursodeoxycholic acid ↓ TNF-α, IL-1β, IL-6, MDA, COX-2, MAO, Tau, 5-HT, Dopamine, Epinephrine, GABA, Glu, Norepinephrine, Deoxycholic acid, Glycochenodeoxycholic acid, Glylilithocholic acid, Lithocholic acid, Taurocholic acid, Taurodeoxycholic acid, Taurolithocholic acid	N.R./China/ Ethanol extract	Leave
*Dengzhan shengmai* [124]	Ischemic stroke	Male SD rats 720 mg/kg 4 weeks PC12 cells	Two-vessel occlusion	↑ Prevotellaceae, Bacteroidaceae, Alloprevotella, Bacteroidetes, Lactobacillus, Allobaculum, Ruminococcus torques, Ruminococcus gauvreauii ↓ Anaerostipes, Monoglobaceae, Monoglobus, Erysipelotrichaceae, Erysipelatoclostridium, Oscillospira, Lachnospiraceae_ND3007, Lachnospiraceae_UCG-004, Candidatus Saccharimonas ↑ SOD, AKT, PI3K, ZO-1, Occludin, MCT1, SMCT1, Acetic acid, Propionic acid, Butyric acid ↓ TNF-α, IL-1β, IL-6, MDA, Caspase, LPS, FABP2	Phenolic acids, Flavonoids, Saponins, Lignans/ China/N.R.	Herba of Erigeron breviscapus, Root of *Panax ginseng*, Fruit of *Schisandra chinensis*, Root of *Ophiopogon japonicus*
Dioscorea polystachya [125]	Ischemic stroke and Diabetes	Male Wistar rats	Streptozotocin MCAO/R	↑ Lactobacillus, Ruminococcus, Clostridium ↑ SOD, GABA, 5-HT, BDNF, Acetic acid, Propionic acid, Butyric acid ↓ TNF-α, IL-1β, MDA, FBG, LPS	Saponin (Diosgenin) Yam polysaccharide, Allantoin, Mucus protein/China/ Water extract	Yam gruel

**Abbreviations**: 5-hydroxyindole acetic acid (5-HIAA), 5‐hydroxytryptamine (5‐HT), Aspartic acid (Asp), Amyloid precursor Protein (APP), Brain-derived neurotropic factor (BDNF), Catalase (CAT), Cyclooxygenase 2 (COX-2), Fatty acid binding protein (FABP), Fasting blood glucose (FBG), Glutamic acid (Glu), Glutathione (GSH), High-density lipoprotein cholesterol (HDL-C), Interleukin (IL), LPS binding protein (LBP), Malondialdehyde (MDA), Middle cerebral artery occlusion/reperfusion (MCAO/R), Monocarboxylate transporter 1 (MCT1), Matrix metalloproteinase-9 (MMP-9), Nitroblue tetrazolium solution (NBT), Nuclear factor κ-light-chain-enhancer of activated B cells (NF-κB), Not reported (N.R.), Phosphoinositide 3-kinase (PI3K), Protein kinase B (AKT), Sodium-coupled monocarboxylate transporter (SMCT1), Superoxide dismutase (SOD), Toll-like receptor 4 (TLR4), Transient forebrain ischemia/ reperfusion (TFI/R), Tumor necrosis factor‐α (TNF‐α), Zonula occludens-1 (ZO-1), γ‐aminobutyric acid (GABA).

## LIST OF ABBREVIATIONS

ACh: Acetylcholine
AChE: Acetylcholinesterase
ACTH: Adrenocorticotropic Hormone
*ALT*: *Alanine Transaminase*
AGIQ: Alpha-glycosyl Isoquercitrin
AD: Alzheimer’s Disease
Aβ: Amyloid Beta
APP/PS1: Amyloid Precursor Protein- Presenilin 1
ALS: Amyotrophic Lateral Sclerosis
APCs: Antigen-presenting Cells
Apaf-1: Apoptosis Protease-activating Factor 1
*AST*: *Aspartate Transaminase*
Bcl-2: B-cell Lymphoma Protein 2,-associated X Bax
BBB: Blood-brain Barrier
BDNF: Brain-derived Neurotrophic Factor
CREB: cAMP-response Element Binding Protein
*CAT*: Catalase
COMT: Catechol-O-methyl Transferase
Cdc42: Cell Division Control Protein 42 Homolog
CNS: Central Nervous System
CSDS: Chronic Social Defeat Stress
*JNKs*: *c-Jun N-terminal Kinases*
CCF: Coptis Chinensis Franch
CORT: *Corticosterone*
CRH: Corticotropin-releasing Hormone
COX-2: Cyclooxygenase-2
DCs: Dendritic Cells
DNMTs: DNA Methyltransferases
ENS: Enteric Nervous System
ERK: Extracellular Signal-regulated Kinase
FAD: Familial AD
*FAT*/*CD36*: Fatty Acid Translocase/Cluster of Differentiation 36
GABA: Gamma-aminobutyric Acid
GF: Germ-free
*GFAP*: *Glial Fibrillary Acidic Protein*
GLP-1: Glucagon-like Peptide-1
GluR1: Glutamate Receptor 1
GluA1: Glutamate Receptor Subunit-1
GSH: Glutathione
*GST*: Glutathione-S-Transferase
GSK-3β: Glycogen Synthase Kinase-3 beta
GPRs: G-protein-coupled Receptors
GALT: Gut-associated Lymphoid Tissue
*HO*: Heme Oxygenase
HDAC: Histone Deacetylase
HPA: Hypothalamic-pituitary-adrenal
iNOS: Inducible Nitric Oxide Synthase
IFN-γ: Interferon-gamma
IL: Interleukin
IECs: Intestinal Epithelial Cells
*Iba1*: Ionized Calcium Binding Adaptor Molecule 1
Kyn: Kynurenine
KEGG: Kyoto Encyclopedia of Genes and Genomes
LC3: Light Chain 3
LBP: Lipopolysaccharide Binding Protein
LPS: Lipopolysaccharides
*LDL*: *Low-density Lipoprotein*
MDA: *Malondialdehyde*
mTOR: Mammalian Target of Rapamycin
MCAO: Middle Cerebral Artery Occlusion
MMP: Mitochondrial Membrane Potential
*MAPKs*: Mitogen-activated Protein Kinases
MCT: Monocarboxylate Transporters
MUC-2: Mucin 2
MS: Multiple Sclerosis
MPO: *Myeloperoxidase*
MPTP: 1-methyl-4-phenyl-1,2,3,6-Tetrahydropyri-dine
*NLRP3*: NOD-like Receptor Protein 3
non-HDL: Non-high-density Lipoprotein
*Nrf2*: Nuclear Factor Erythroid 2-Related Factor 2
N.R.: Not reported
*NF*-*κB*: Nuclear Factor Kappa B
*NOD*: Nucleotide-binding and Oligomerization Domain
NODs: Nucleotide-binding Oligomerization Domain Molecules
PD: Parkinson’s Disease
PAMPs: Pathogen-associated Molecular Patterns
PRRs: Pattern Recognition Receptors
PPAR-γ: Peroxisome Proliferator-activated Receptor Gamma
PI3K: Phosphatidylinositol 3 Kinase, Poly(ADP-ribose)
PARP: Polymerase
PSD95: Postsynaptic Density Protein 95
PEP: Prolyl Endopeptidase
RNS: Reactive Nitrogen Species
ROS: Reactive Oxygen Species
Tregs: Regulatory T Cells
5-HT: Serotonin
SCFAs: Short-chain Fatty Acids
SIRT1: Sirtuin 1
SOD: Superoxide Dismutase
TIMP3: Tissue Inhibitors of Metalloproteinase 3
*TNFR*1: *TNF Receptor*‐1
TRAIL: TNF-related Apoptosis-inducing Ligand
TLRs: Toll-like Receptors
TGF-β: Transforming Growth Factor-β
TrkB: Tropomyosin Receptor Kinase B
TPH2: Tryptophan Hydroxylase 2
*TNF*-*α*: *Tumor Necrosis Factor*-*alpha*
VOZB: Zanthoxylum Bungeanum
ZZCD: Zhi-Zi-Chi
*ZO*-*1*: Zonula Occludens-1

## STANDARDS OF REPORTING

PRISMA guidelines and methodology were followed.

## SUPPLEMENTARY MATERIAL

PRISMA checklist is available as supplementary material on the publisher’s website along with the published article.
